# Pro-Inflammatory Cytokine Regulation of P-glycoprotein in the Developing Blood-Brain Barrier

**DOI:** 10.1371/journal.pone.0043022

**Published:** 2012-08-13

**Authors:** Majid Iqbal, Hay Lam Ho, Sophie Petropoulos, Vasilis G. Moisiadis, William Gibb, Stephen G. Matthews

**Affiliations:** 1 Department of Physiology, University of Toronto, Toronto, Ontario, Canada; 2 Department of Obstetrics and Gynecology, University of Ottawa, Ottawa, Ontario, Canada; 3 Department of Cellular and Molecular Medicine, University of Ottawa, Ottawa, Ontario, Canada; 4 Department of Obstetrics and Gynecology, University of Toronto, Toronto, Ontario, Canada; 5 Department of Medicine, Faculty of Medicine, University of Toronto, Toronto, Ontario, Canada; Université de Montréal, Canada

## Abstract

Placental P-glycoprotein (P-gp) acts to protect the developing fetus from exogenous compounds. This protection declines with advancing gestation leaving the fetus and fetal brain vulnerable to these compounds and potential teratogens in maternal circulation. This vulnerability may be more pronounced in pregnancies complicated by infection, which is common during pregnancy. Pro-inflammatory cytokines (released during infection) have been shown to be potent inhibitors of P-gp, but nothing is known regarding their effects at the developing blood-brain barrier (BBB). We hypothesized that P-gp function and expression in endothelial cells of the developing BBB will be inhibited by pro-inflammatory cytokines. We have derived brain endothelial cell (BEC) cultures from various stages of development of the guinea pig: gestational day (GD) 50, 65 (term ∼68 days) and postnatal day (PND) 14. Once these cultures reached confluence, BECs were treated with various doses (10^0^–10^4 ^pg/mL) of pro-inflammatory cytokines: interleukin-1β (IL-1β), interleukin-6 (IL-6) or tumor necrosis factor- α (TNF-α). P-gp function or *abcb1* mRNA (encodes P-gp) expression was assessed following treatment. Incubation of GD50 BECs with IL-1β, IL-6 or TNF-α resulted in no change in P-gp function. GD65 BECs displayed a dose-dependent decrease in function with all cytokines tested; maximal effects at 42%, 65% and 34% with IL-1β, IL-6 and TNF-α treatment, respectively (P<0.01). Inhibition of P-gp function by IL-1β, IL-6 and TNF-α was even greater in PND14 BECs; maximal effects at 36% (P<0.01), 84% (P<0.05) and 55% (P<0.01), respectively. Cytokine-induced reductions in P-gp function were associated with decreased *abcb1* mRNA expression. These data suggest that BBB P-gp function is increasingly responsive to the inhibitory effects of pro-inflammatory cytokines, with increasing developmental age. Thus, women who experience infection and take prescription medication during pregnancy may expose the developing fetal brain to greater amounts of exogenous compounds – many of which are considered potentially teratogenic.

## Introduction

The developing fetus is protected from potentially teratogenic compounds in the maternal circulation by multidrug resistance transporter proteins, such as P-glycoprotein (P-gp). At the placenta, P-gp acts to extrude a wide range of xenobiotics from the syncytiotrophoblast and away from the fetal compartment. Placental expression of P-gp has been shown in many mammalian species, including humans, to be high early in pregnancy before decreasing with advancing gestation [1]–[4]. Functionally, this decreased expression leads to increased accumulation of P-gp substrates in the fetus and amniotic fluid [5]. The decline in global fetal protection provided by placental P-gp leaves the fetus susceptible to teratogens that may be in maternal circulation. Epidemiological studies in various countries have estimated that 64–96% of women take prescription medication during pregnancy; 5–10% of which are considered to be potentially teratogenic [6]–[11]. Many of these drugs are P-gp substrates [12]–[14]. Exposure to environmental pollutants (including pesticides and herbicides) is also increasing, leading to increased incidence of teratogenesis, especially in developing industrialized countries [15]–[17]. Again many of these substances are substrates for P-gp [12].

We (and others) have shown the developing fetal blood-brain barrier (BBB) compensates for the declining placental protection by dramatically increasing the expression of BBB P-gp, near-term; increasing local brain protection [18]–[20]. However, as this chemical barrier develops at the fetal BBB, the developing fetal brain remains vulnerable to circulating xenobiotics that can pass through the placenta as a result of the decline in placental P-gp.

Fetal brain vulnerability may be even more pronounced in pregnancies complicated by maternal and/or intra-amniotic infection. Bacterial and viral infection is common during pregnancy [21], [22] and accounts for approximately 40% of preterm births [23]. Further, in developing countries, parasitic infections such as malaria [24] and toxoplasmosis [25] have detrimental effects on the developing fetus. Rodent models of infection involving lipopolysaccharide (LPS; a model of bacterial infection) or polyinosinic-polycytidylic acid (a model of viral infection) injections during pregnancy have demonstrated reductions in placental *abcb1a* and *abcb1b* mRNA (translated into P-gp) expression, suggesting reduced fetal protection against xenobiotics [2], [26], [27]. During an infection pro-inflammatory cytokines are released from endothelial cells and various cells of the immune system [27]–[34]. Pro-inflammatory cytokines have been shown to potently inhibit P-gp. *In vivo* studies involving the injection of adult mice with pro-inflammatory cytokines, interleukin-1β (IL-1β), interleukin-6 (IL-6) or tumor necrosis factor-α (TNF-α), results in significant decreases in P-gp mRNA expression in the liver [35]. Similar inhibition of P-gp expression and function has also been reported in adult rat hepatocytes and brain endothelial cells (BECs) following exposure to IL-1β, IL-6 or TNF-α, *in vitro* [36]–[41]. However, nothing is known as to how infection and pro-inflammatory cytokines can alter P-gp expression at the developing fetal BBB.

In this study, we examined the effects of pro-inflammatory cytokines on P-gp function in BECs derived at various stages of fetal development. We have previously established and characterized a robust BEC culture system in which we have derived BECs from various stages of development – both fetal and postnatal [19]. These BECs have clearly been demonstrated to maintain a distinct P-gp functional phenotype, which corresponds to their developmental age [19]. We hypothesized that treatment with IL-1β, IL-6 or TNF-α will result in decreased P-gp function in BECs, and that this inhibitory effect will increase in magnitude with increasing developmental age. These data are critical to increasing our understanding of how infection-complicated pregnancies can potentially harm the developing fetal brain.

## Results

### Developmental Changes in Baseline P-gp Function

**Figure 1:**
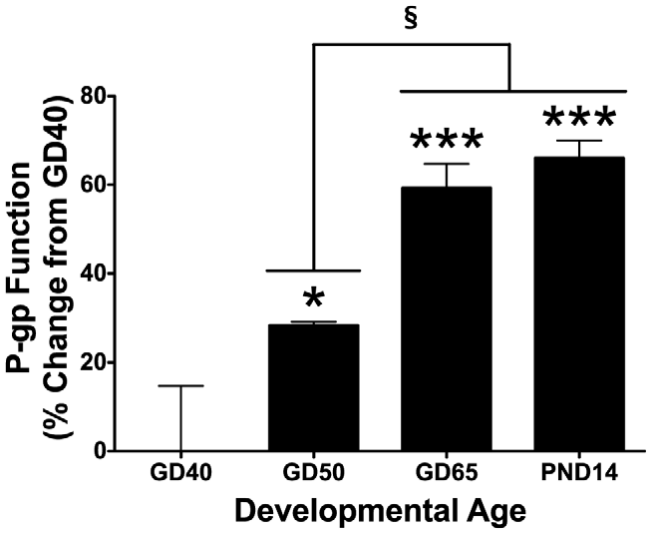
Baseline P-gp Activity in BECs increases with developmental age. P-glycoprotein (P-gp) activity in brain endothelial cell (BEC) cultures derived from gestational day (GD) 40, 50, 65 and postnatal day (PND) 14 male guinea pigs (N = 4). P-gp activity is displayed as percent change from GD40 BECs (zero line). P-gp function was calculated over relative cell count. Values displayed as mean ± S.E.M. A significant difference from GD40 indicated by (*) P<0.05; (***) P<0.001.

BECs derived from male guinea pigs were utilized in this study; we have previously identified no sex-differences in the developmental expression of P-gp protein [19]. Compared to BECs derived from GD40, GD50 BECs exhibited approximately 28% more P-gp function (P<0.05; Fig. 1). Likewise, BECs derived from GD65 and PND14 male guinea pigs displayed significantly increased P-gp function compared to GD40 BECs (59% and 66% increases, respectively; P<0.001) and GD50 BECs (P<0.05). There was no significant difference in P-gp function between BECs derived from GD65 and PND14.

### Developmental Changes in Cytokine-Responsiveness

**Figure 2:**
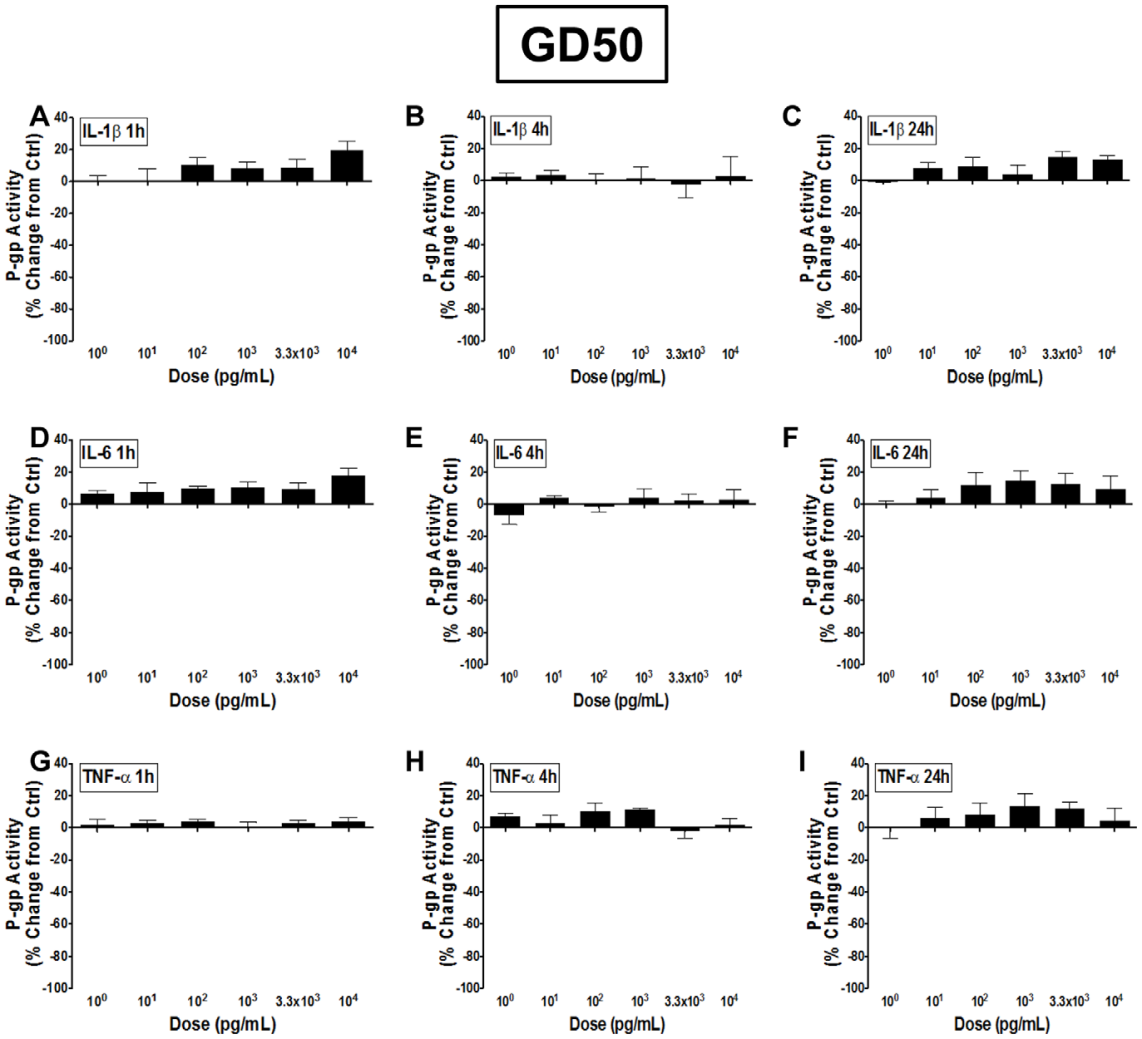
P-gp function in GD50 BECs is unresponsive to pro-inflammatory cytokines. P-glycoprotein (P-gp) activity in brain endothelial cell (BEC) cultures derived from gestational day (GD) 50 male guinea pigs following treatment with 10^0^–10^4^ pg/mL A–C) interleukin-1β (IL-1β), D–F) interleukin-6 (IL-6) or G–I) tumor necrosis factor-α (TNF-α) for 1, 4 or 24 hours (N = 4). P-gp activity is displayed as percent change from untreated control cells (zero line). Values displayed as mean ± S.E.M.

Male guinea pig BECs derived from GD50 exhibited little to no change in P-gp function to any dose of IL-1β, IL-6 or TNF-α, when treated for 1, 4 or 24 hours, *in vitro* (Fig. 2). There was a slight tendency for an increase in P-gp function, though this did not reach significance for any of the cytokines tested.

**Figure 3:**
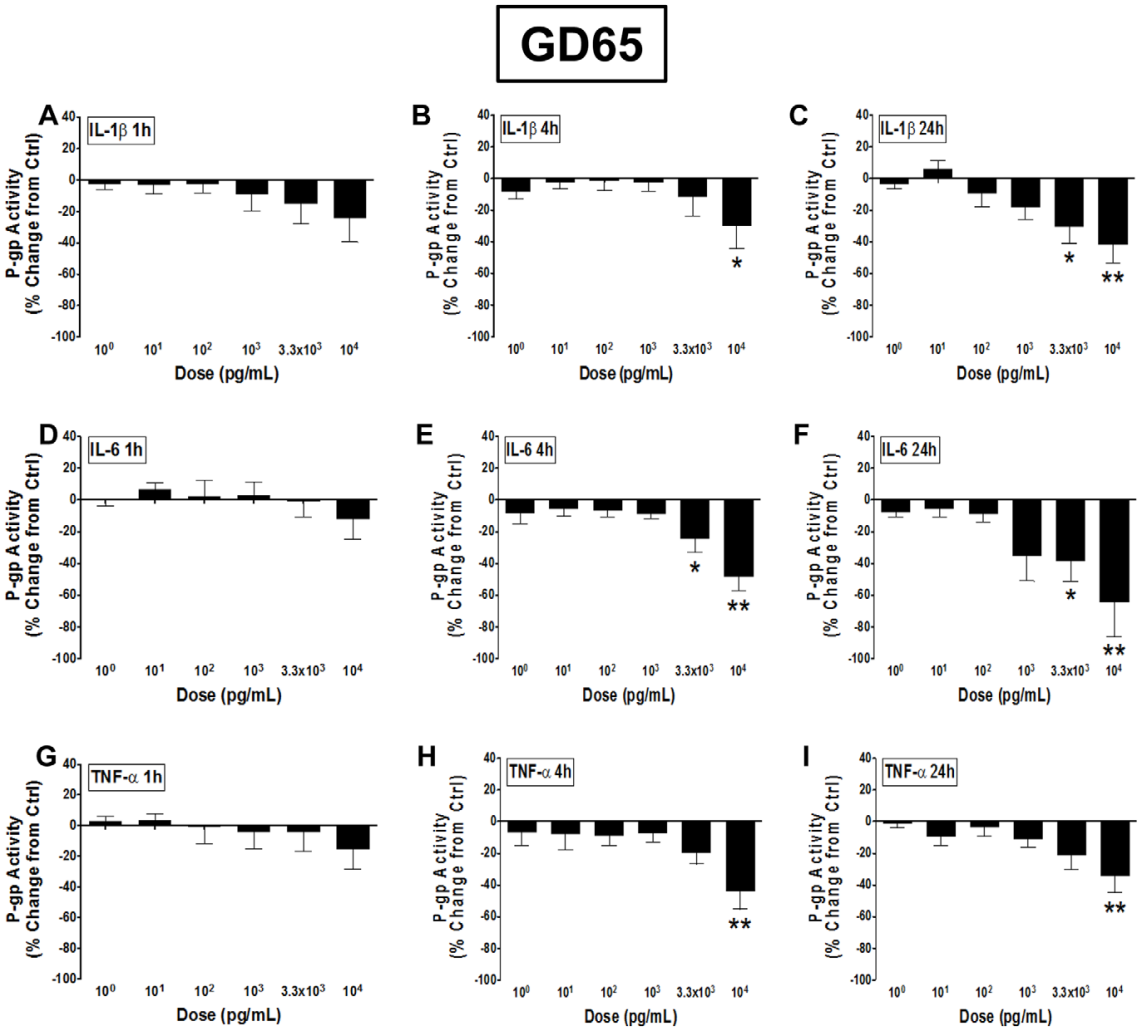
Pro-inflammatory cytokines inhibit P-gp function in GD65 BECs in a dose-dependent manner. P-glycoprotein (P-gp) activity in brain endothelial cell (BEC) cultures derived from gestational day (GD) 65 male guinea pigs following treatment with 10^0^–10^4^ pg/mL A–C) interleukin-1β (IL-1β), D–F) interleukin-6 (IL-6) or G–I) tumor necrosis factor-α (TNF-α) for 1, 4 or 24 hours (N = 4). P-gp activity is displayed as percent change from untreated control cells (zero line). Values displayed as mean ± S.E.M. A significant difference from control indicated by (*) P<0.05; (**) P<0.01.

When treated with IL-1β for 1 hour, BECs derived from GD65 male guinea pigs displayed a trend towards a decrease in function, particularly with higher doses (Fig. 3A). Following 4 hours of treatment the 10^4^ pg/mL dose of IL-1β resulted in a significant decrease in P-gp function (approximately 30% inhibition; P<0.05; Fig. 3B). This IL-1β-induced inhibition was even greater following 24 hours of treatment, displaying a dose-dependent decrease in P-gp function with 3.3×10^3^ (∼30%; P<0.05) and 10^4^ (∼42%; P<0.01) pg/mL doses (Fig. 3C). Incubation with IL-6 for 1 hour resulted in no change in P-gp function in GD65 BECs (Fig. 3D), however 4-hour treatment resulted in a dose-dependent decrease in function at 3.3×10^3^ (∼25%; P<0.05) and 10^4^ (∼48%; P<0.01) pg/mL doses (Fig. 3E). At these same doses, inhibition was even greater following 24 hours of IL-6 treatment of GD65 BECs –38% (P<0.05) and 65% (P<0.01), respectively (Fig. 3F). Only the highest dose of TNF-α (10^4^ pg/mL) treatment of GD65 BECs only resulted in inhibition of P-gp function at 4 (∼44%; P<0.01; Fig. 3H) and 24 hours (∼34%; P<0.01; Fig. 3I).

**Figure 4:**
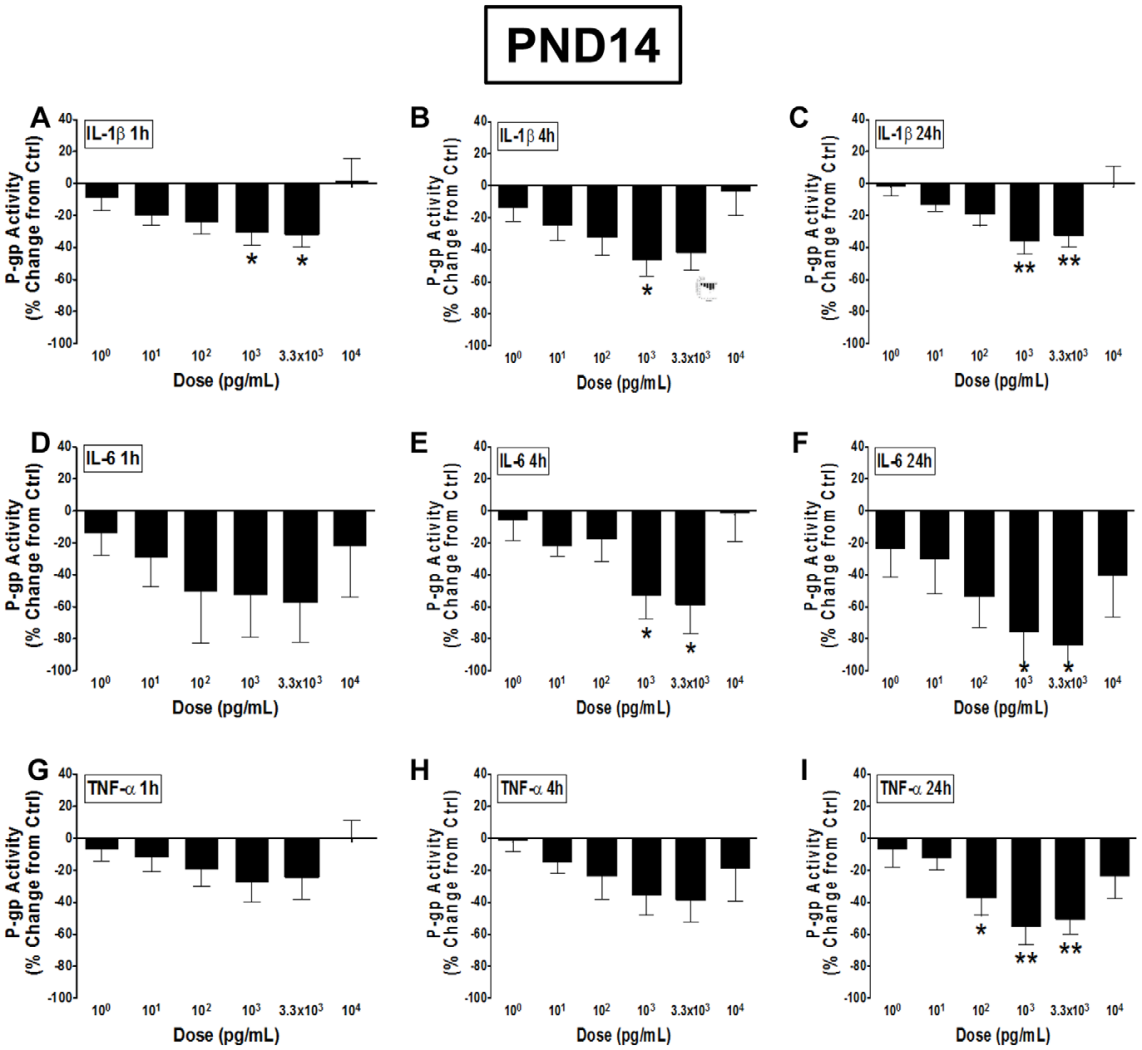
The inhibitory effects of pro-inflammatory cytokines on P-gp function are greatest in PND14 BECs. P-glycoprotein (P-gp) activity in brain endothelial cell (BEC) cultures derived from postnatal day (PND) 14 male guinea pigs following treatment with 10^0^–10^4^ pg/mL A–C) interleukin-1β (IL-1β), D–F) interleukin-6 (IL-6) or G–I) tumor necrosis factor-α (TNF-α) for 1, 4 or 24 hours (N = 8). P-gp activity is displayed as percent change from untreated control cells (zero line). Values displayed as mean ± S.E.M. A significant difference from control indicated by (*) P<0.05; (**) P<0.01.

The inhibitory effects of cytokines on P-gp function were even greater on BECs derived from PND14. Treatment with 10^3^ and 3.3×10^3^ pg/mL IL-1β for 1 hour resulted in 31% and 32% decrease in function, respectively (Fig. 4A). This trend continued following 4 (P<0.05) and 24 (P<0.01) hour incubations with IL-1β (Fig. 4B–C). PND14 BECs following treatment with IL-6 exhibited the strongest inhibition of P-gp function. IL-6 treatment for 1 hour resulted in a trend towards decreased P-gp function, though this did not reach significance due to high variability (Fig. 4D). Following 4 hours of incubation, P-gp function decreased 53% and 59% with the 10^3^ (P<0.05) and 3.3×10^4^ (P<0.05) pg/mL dose of IL-6, respectively (Fig. 4E). The inhibition reached 76% and 84% at these same respective doses of IL-6, when incubated for 24 hours (P<0.05; Fig. 4F). Treatment of PND14 BECs with TNF-α for 1 and 4 hours resulted in a trend towards a dose-dependent inhibition of P-gp function, although these did not reach significance (Fig. 4G–H). Following 24 hours incubation of 10^2^, 10^3^ and 3.3×10^3^ pg/mL doses of TNF-α, P-gp function was decreased by 38% (P<0.05), 55% (P<0.01) and 50% (P<0.01), respectively (Fig. 4I). There was little to no change in P-gp function following treatment with the highest (10^4^ pg/mL) dose of IL-1β, IL-6 or TNF-α.

### Specificity of Pro-inflammatory Cytokine Effects on P-gp Activity

**Figure 5:**
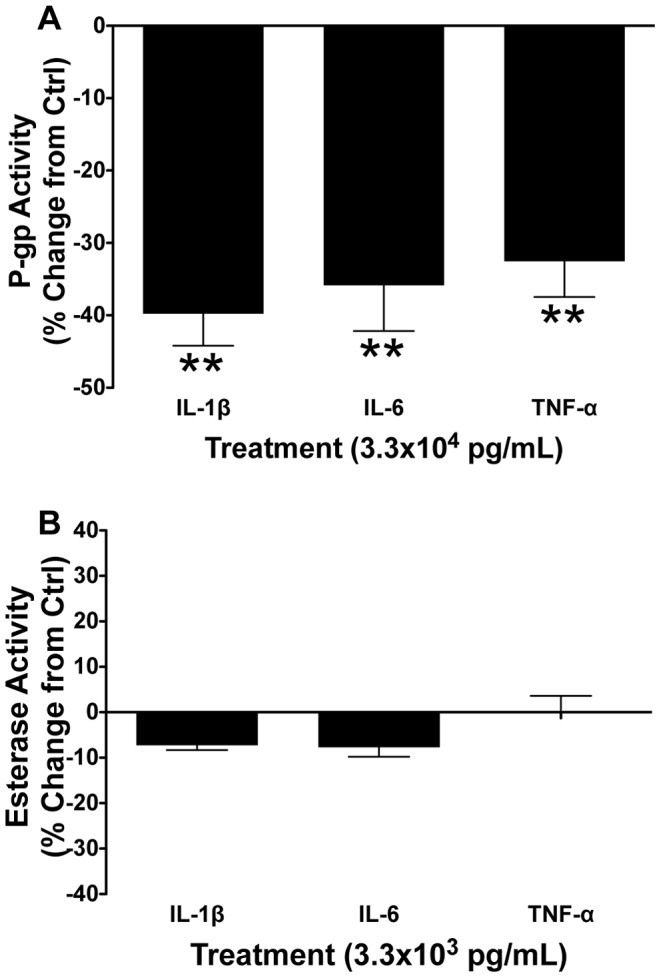
The inhibitory effects of pro-inflammatory cytokines are specific to P-gp and do not alter non-specific esterase activity. Postnatal day 14 male guinea pig BEC A) P-gp activity (using Rhodamine 123 as a P-gp substrate) following 24 hour treatment with 3.3×10^3^ pg/mL interleukin-1β (IL-1β), interleukin-6 (IL-6) or tumor necrosis factor-α (TNF-α) (N = 6); and B) non-specific esterase activity following 24 hour treatment with 3.3×10^3^ pg/mL IL-1β, IL-6 or TNF-α (N = 6). P-gp activity is displayed as percent change from untreated control cells (zero line). Values displayed as mean ± S.E.M. A significant difference from control indicated by (**) *P*<0.01.

The alternative P-gp substrate, Rho123, was used to confirm that the cytokine-induced effects on P-gp function were in fact P-gp specific. IL-1β, IL-6 or TNF-α treatments resulted in decreased Rho123 accumulation (and thus increased P-gp function; *P*<0.01; Fig. 5A) – similar to the inhibition of P-gp function seen in experiments using calcein-AM.

Calcein-AM is converted to fluorescent calcein by cytoplasmic non-specific esterases, in addition to extrusion by P-gp. To determine if the conversion of calcein-AM to calcein was affected by cytokine treatment, changes in esterase activity were assessed. Treatment of BECs derived from PND14 guinea pigs with 3.3×10^3^ pg/mL of IL-1β, IL-6 or TNF-α for 24 hours resulted in no change in esterase activity, compared to controls (P>0.05; Fig. 5B).

### Changes in Abcb1 mRNA Expression Following Cytokine Treatment

**Figure 6:**
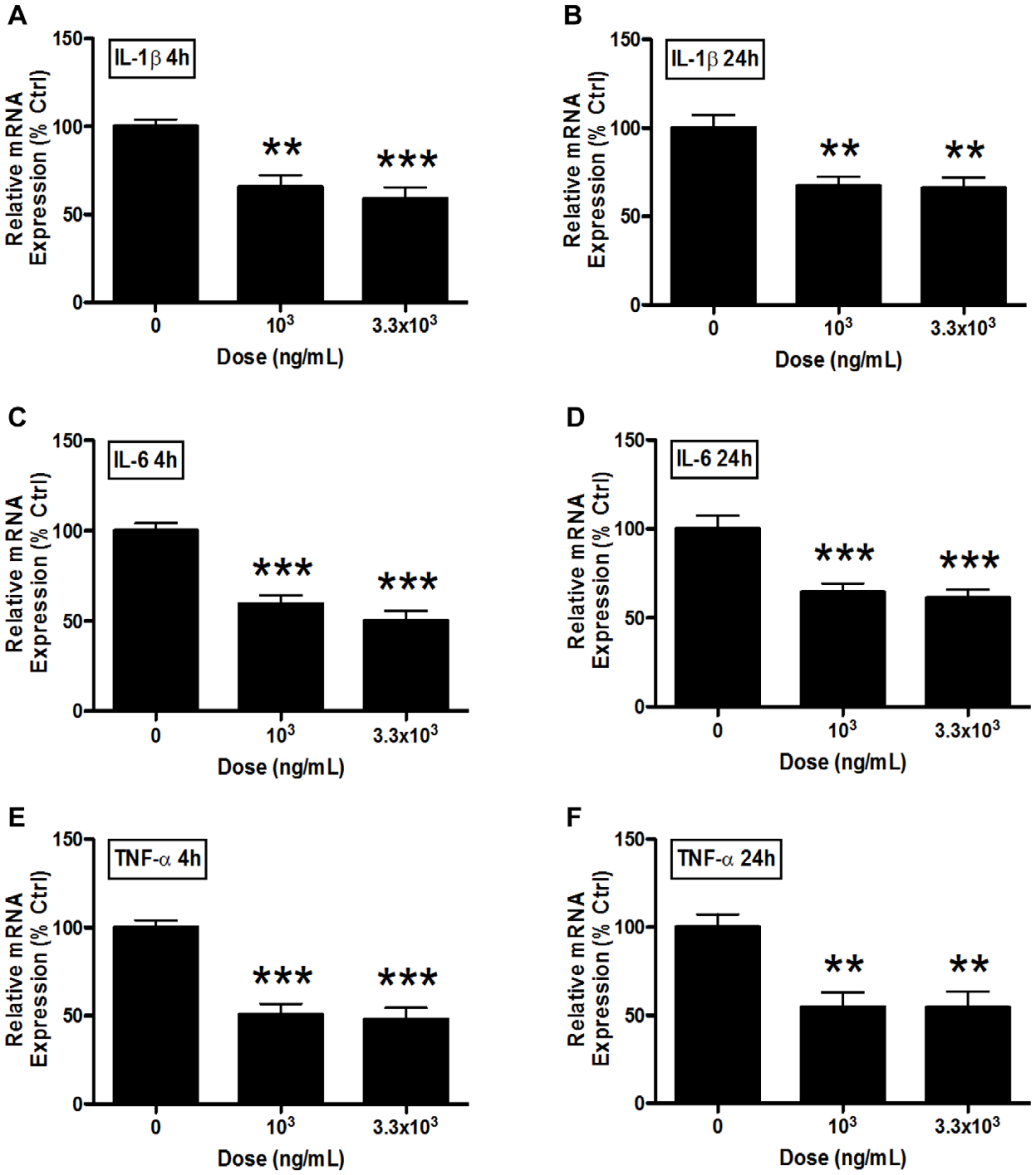
Pro-inflammatory cytokines inhibit *abcb1* mRNA expression. *Abcb1* mRNA expression in brain endothelial cell (BEC) cultures derived from postnatal day (PND) 14 male guinea pigs following treatment with 10^3^ and 3.3×10^3^ pg/mL A–B) interleukin-1β (IL-1β), C–D) interleukin-6 (IL-6) or E–F) tumor necrosis factor-α (TNF-α) for 4 (A,C,E) or 24 hours (B,D,F) (N = 7–8). Expression is displayed as percent of untreated control cell expression (i.e. 100% line) and taken over the reference gene, *beta-actin*. Values displayed as mean ± S.E.M. A significant difference from control indicated by (**) P<0.01; (***) P<0.001.

Treatment of PND14 BECS with 10^3^ and 3.3×10^3^ pg/ml of IL-1β for 4 hours resulted in a 34% (P<0.01) and 41% (P<0.001) decrease in *abcb1* mRNA, respectively (Fig. 6A). This decreased expression is present following 24 hours of treatment as well, at roughly 33% (10^3 ^pg/mL; P<0.01) and 34% (3.3×10^3^ pg/mL; P<0.01) (Fig. 6B). Similar decreases in *abcb1* mRNA expression occur following IL-6 and TNF-α treatment. IL-6 displayed strong inhibition that ranged from 41–50% and 36–39% decreases in expression at 4 and 24 hours, respectively (P<0.001; Fig. 6C–D). TNF-α treatment for 4 and 24 hours resulted in 49–52% (P<0.001) and 45–46% inhibition (P<0.01), respectively (Fig. 6E–F).

## Discussion

We have demonstrated for the first time that pro-inflammatory cytokines exhibit an inhibitory effect on P-gp function in endothelial cells derived from the developing BBB. Our results indicate that the magnitude of this inhibition increases substantially with advancing age.

We have previously shown that P-gp protein expression in the developing BBB of the guinea pig increases approximately 7 to 8-fold between GD50 and GD65, and 14 to 17-fold between GD50 and PND14 [19]. As found in this current study, these changes in P-gp protein correspond to a 31% (at GD65) and 38% (at PND14) increase in *in vitro* BEC P-gp function, respectively when compared to GD50 BECs. This change in P-gp expression and function correspond with previous studies in mice and rats, which displayed increased developmental expression of P-gp in the whole brain [18], [20], as well as decreased *in vivo* radiolabelled P-gp substrate accumulation in the fetal brain with advancing gestation [20]. Unfortunately, it is not possible to assess in P-gp function in guinea pigs, *in vivo*, due to their large size (i.e. pregnant guinea pigs can be 20–25X greater in size than a pregnant mouse) and therefore the prohibitive costs of undertaking radiolabelled substrate accumulation studies. Functional studies of fetal human BBB activity are also not possible due to unavailability of tissues. However, human studies have shown increases in fetal brain P-gp expression with advancing gestation [42]–[44], suggesting that brain protection increases with developmental age, as we find in our and other rodent studies.

Our data suggest that while P-gp protection is developing at the fetal BBB, infection may render the developing brain susceptible to circulating xenobiotics and teratogens. Our experiments in BECs derived from PND14 clearly indicate that pro-inflammatory cytokines, IL-1β, IL-6 and TNF-α, have strong inhibitory effects on P-gp function. These results are in accordance with previous studies conducted in adult rodent hepatocytes and brain endothelial cells [36]–[40]. However, we have identified, for the first time that the inhibitory effects of pro-inflammatory cytokines on BBB P-gp function extend into fetal and neonatal life. In the fetal guinea pig, responsiveness to these inhibitory effects appears to develop between GD50 and term (∼GD68). At least at the level of the BBB, it appears that prior to GD50 pro-inflammatory cytokine release due to infection does not act to inhibit P-gp function. A lack of fetal BBB P-gp inhibition by pro-inflammatory cytokines up to 75% through gestation may bode well for pregnant women who experience infection earlier in pregnancy. At the same time, BBB P-gp expression and function is relatively low prior to GD50 (compared to term) and therefore may be much less adequate at protecting the fetal brain – with or without inhibition by pro-inflammatory cytokines. It is important to note that placental P-gp is also inhibited by infection [2], [26], [27], creating the potential for even greater exposure of the developing fetal brain to xenobiotics during maternal infection. Further *in vivo* studies are critical to elucidating the protective capacity of the fetal brain at various gestational time points, and how infection and/or pro-inflammatory cytokine exposure can alter fetal brain protection. It also remains unknown whether pro-inflammatory cytokine inhibition of P-gp function continues in brain endothelial cells past PND14. However, previous studies have found similar inhibitory effects of pro-inflammatory cytokines on adult (rodent and human) brain endothelial cell multidrug resistance [40], [41], suggesting that this responsiveness persists into adult life. As such, an infection in postnatal life through to adulthood may well reduce the protective capacity of the blood-brain barrier.

The intracellular mechanisms involved in the pro-inflammatory cytokine-induced reductions in P-gp function and expression remain unknown. Many regulatory pathways typically activated by pro-inflammatory cytokines, such as nuclear factor kappa B (NF-κB) [45], [46], activator protein-1 (AP-1) [47] and signal transducer and activator of transcription (STAT) [48], have been associated with the modulation of P-gp function and expression [45], [47]–[52]. The human *ABCB1* promoter contains binding elements for NF-κB, AP-1 and STAT, suggesting the direct involvement of these regulatory proteins in the modulation of *ABCB1* expression [53]–[56]. These regulatory protein-gene promoter interactions need to be further investigated in future studies, particularly whether or not they are involved in the alteration of multidrug resistance of guinea pig brain endothelial cells.

The increase in fetal BEC responsiveness to the inhibitory effects of pro-inflammatory cytokines (at the level of P-gp function) in late gestation suggests that fetal brain protection is potentially most vulnerable to infection at this time. Baseline P-gp function increases 31% between GD50 and GD65, but pro-inflammatory cytokine treatment of GD65 BECs results in a 30–50% decrease in function – essentially negating (and further inhibiting) the rise in baseline P-gp activity. This finding must be confirmed with *in vivo* models of infection near term, but certainly raises concerns for mothers experiencing infection in late gestation. Clinically, a pregnant women presenting with maternal or intra-amniotic infection is at risk of preterm labor (PTL) [23], and these women are often administered pharmacological treatments. Approximately 10% of women receive synthetic glucocorticoid treatment for being at risk of PTL (whether they present with infection or not) to help mature the fetal lungs and reduce the incidence of infant respiratory distress syndrome [57], [58]. Though this treatment has greatly improved neonatal outcomes, it has been associated with many long-term health consequences [59] and we have shown synthetic glucocorticoids to be potent modulators of fetal BEC P-gp activity [19]. Synthetic glucocorticoids such as dexamethasone and betamethasone are also substrates of P-gp, thus inhibition of fetal BBB P-gp by pro-inflammatory cytokines can potentially allow more synthetic glucocorticoid into the brain. Clearly, the clinical situation of preterm labor and synthetic glucocorticoid administration appears to be multi-faceted and thus it is critical to understand how this common clinical practice can alter fetal BBB multidrug resistance development, particularly in conjunction with infection. Often hormones such as progesterone, antibiotics or tocolytics are given to mothers at risk of PTL in order to prevent or delay labor [60]–[62]. However, many of these compounds are P-gp substrates [12], [63]–[65] and little to nothing is known regarding their long-term (potentially teratogenic) neurological effects in the fetus and newborn. Our data suggest that greater caution may be necessary when administering treatments or prescription medication to pregnant women with infections, but further study is critical to understand the effects on BBB multidrug resistance development and long-term consequence on the health of the newborn.

The rise in P-gp expression [19] and function, as well the increase in BEC responsiveness to cytokines may be attributed to epigenetic changes at the level of the *abcb1* gene promoter as well as the endogenous late gestation surge in maternal and fetal plasma glucocorticoids. Changes in promoter methylation of the *abcb1* gene at these developmental time points may explain the increased P-gp expression/function that we have identified, as well as help explain the increased ability of pro-inflammatory cytokine pathways to modulate transcription – though this remains speculative. Our previous data suggest that the late gestation glucocorticoid surge may represent an important signal for the upregulation of P-gp protein, function and responsiveness to pro-inflammatory cytokines at the fetal BBB, as this surge is critical to the maturation of many fetal organs including the lungs and brain [59], [66], and likely the BBB. Future studies are necessary to investigate whether glucocorticoids and/or epigenetic processes play a role in the increased protective capacity and responsiveness to pro-inflammatory cytokines at the developing BBB.

In conclusion, this is the first study to identify the effects of pro-inflammatory cytokines on multidrug resistance in the developing BBB. Our results clearly indicate that cytokine-induced inhibition of P-gp function increase in magnitude with advancing gestation. These changes in fetal BEC responsiveness to pro-inflammatory cytokines certainly warrant further mechanistic investigation. Indeed, these studies are critical given the high incidence of maternal and intra-amniotic infection and the large number of pregnant women on medication during pregnancy. Understanding how events, such as infection, during gestation can negatively affect the drug susceptibility of the offspring will be critical in the development of future therapies to counteract these effects.

## Materials and Methods

### Animals and Breeding

The guinea pig was chosen as the model organism due to its comparably longer gestation (approximately 68 days, compared to 19 and 21 days for the mouse and rat, respectively), which allowed for a wider range of developmental time points from which BECs could be derived. Guinea pigs give birth to neuroanatomically mature young, and undergo a pattern of fetal brain development that closely resembles humans and primates [67]. Placentation in the guinea pig is also similar to humans. Twelve week-old nulliparous female and postnatal day (PND) 14 male Dunkin-Hartley-strain guinea pigs were obtained from Charles River Canada Inc. (St. Constant, Quebec, Canada). Twelve week-old females were bred (as described previously [19]), and left untreated during pregnancy. All studies were carried out in accordance with protocols approved by the Animal Care Committee at the University of Toronto and in accordance with the Canadian Council on Animal Care.

### Primary Guinea Pig Microvessel Extraction and BEC Culture

Pregnant guinea pigs were anaesthetized using isoflurane (Baxter Corp., Mississauga, Ontario, Canada) and euthanized by decapitation on gestational day GD40, 50 or 65 (term approximately 68 days; n = 4–6 for each gestational age). From each litter, fetal brains were collected as described previously [19]. Sex of each fetus was determined by the presence of uterine horns or testes. PND14 male guinea pig brains were also collected. Brains were placed on ice-cold Medium 199 (Invitrogen, Carlsbad, California, USA) supplemented with antibiotics-antimycotics (Invitrogen), immediately transferred to a biological safety cabinet and into ice-cold sterile Medium 199 supplemented with antibiotics-antimycotics. All remaining steps took place under sterile conditions. Brains were homogenized, and the homogenate was centrifuged (1000 g, 5 min, 4°C). The pellet was resuspended in dextran (Sigma, St. Louis, Missouri, USA) solution (17.5% w/v dextran in Hank’s Balanced salt solution [Invitrogen]) and centrifuged (4200 g, 15 min, 4°C). Vessel fractions were digested in type 1 collagenase (1 mg/mL; Sigma) dissolved in Dulbecco’s Modified Eagle Medium (DMEM, 15 min, 37°C; Wisent Inc., St. Bruno, Quebec, Canada). Following digestion, the mixture was centrifuged (500 g, 10 min, 4°C), and the cells (pellet) were resuspended in warm DMEM supplemented with 20% fetal bovine serum (Wisent) and ingredients modified from Zhang *et al.* (2003) [68]. Cells were plated on 0.5% gelatin (Sigma)-coated tissue culture flasks (Becton-Dickinson Biosciences, Franklin Lakes, New Jersey, USA) and grown in a 37°C/5% CO_2_-incubator. Following the extraction process, cells were determined to be more than 99% viable by trypan blue staining. We have previously shown that this extraction results in highly pure cultures of BECs (>99%) [19]. BECs derived from male guinea pigs were utilized in this study; we have previously identified no sex-differences in the developmental expression of P-gp protein [19].

### Assessment of Baseline P-gp Function

BECs derived from GD40, 50, 65 and PND14 male guinea pigs were transferred to 0.5% gelatin-coated 96-well plates (Becton-Dickinson; 10,000 cells/well). At confluence, culture medium was removed and cells were washed with warm Tyrode salts’ (Tyrode) solution (Sigma) supplemented with sodium bicarbonate (1 g/L; Sigma). Cells were then incubated for 1 h with calcein-acetoxymethyl ester (calcein-AM; 10^−6^ M; Sigma, St. Louis, Missouri, USA) and C_12_-resazurin (10^−5^ M; Invitrogen) in Tyrode solution. Calcein (cleaved from calcein-AM by endogenous esterases inside the cell) accumulation is commonly used for the assessment of P-gp function in cell-based assays [69], [70]. Resazurin (metabolized into resorufin in live cells) has been used previously for determining relative cell counts [71] – which was used in this study to normalize calcein accumulation measurements in order to compare BEC P-gp function between developmental ages. Resazurin and resorufin are not P-gp substrates, and have previously been used in conjunction with the assessment of P-gp function [72], [73]. Cells were immediately placed on ice following calcein-AM/C_12_-resazurin incubation, washed with ice-cold Tyrode solution and lysed using ice-cold 1% Triton X-100 lysis buffer. Accumulation of fluorescent calcein was determined using a fluorescent plate reader (Calcein: Ex/Em = 485/510 nm; Resorufin: Ex/Em = 540/585 nm; Biotek, Winooski, Vermont, USA). Mean background fluorescence from each plate was subtracted from each control and treated well, prior to analysis, for all experiments measuring P-gp activity.

### Pro-inflammatory Cytokine Effects on P-gp Function

BECs derived from GD50, 65 and PND14 male guinea pigs were transferred to 0.5% gelatin-coated 96-well plates (Becton-Dickinson; 10,000 cells/well). GD40 BECs were not used for cytokine treatment experiments, as we have previously shown that GD40 cells have very low P-gp protein levels and displayed no responsiveness to potent regulators of P-gp (i.e. corticosteroids) [19]. At confluence, culture medium was removed and replaced with phenol red-free DMEM (Wisent) supplemented with 20% charcoal-stripped fetal bovine serum (Wisent). Twenty-four hours following media change, cells were treated with various doses (10^0^–10^4 ^pg/mL) of pro-inflammatory cytokines: IL-1β (Invitrogen), IL-6 (Invitrogen) and TNF-α (Invitrogen) for 1, 4 or 24 h at 37°C. These cytokines have previously been shown to alter P-gp expression and function in rat hepatocyte and rat/porcine BEC cultures [36]–[38], [40], as well as P-gp expression in mouse livers when injected, *in vivo* [35]. Dose ranges correspond to those used in previous studies [36]–[38], [40], as well as levels of circulating cytokines found in human and rodent infection studies [74], [75]. For instance, LPS treatment of post-labor maternal and umbilical cord blood has been shown to result in large elevations in IL-1β (control: ∼1 pg/mL; LPS: ∼150 pg/mL), IL-6 (control: ∼10 pg/mL; LPS: ∼2300 pg/mL) and TNF-α (control: ∼4 pg/mL; LPS: 250 pg/mL) [74]. This study did not investigate the additional cytokine contributions from white blood cells in lymph, as well as from endothelial cells Treatment of BECs derived from PND14 and fetal ages with IL-1β, IL-6 or TNF-α did not result in significant cell death (>99% viability). Control cells were re-incubated with media containing charcoal-stripped fetal bovine serum. Following treatment, cells were washed with warm Tyrode solution and then incubated for 1 h with calcein-AM (10^−6^ M in Tyrode solution). Cells were immediately placed on ice following calcein-AM incubation, washed with ice-cold Tyrode solution and lysed using ice-cold 1% Triton X-100 lysis buffer. Accumulation of fluorescent calcein was determined using a fluorescent plate reader, as described above. Mean background fluorescence from each plate was subtracted from each control and treated well, prior to analysis, for all experiments measuring P-gp activity.

### Pro-inflammatory Cytokine Treatment and P-gp Specificity

BECs derived from PND14 male guinea pigs were grown to confluence in 96-well plates, as described above. At confluence, culture medium was removed and replaced with phenol red-free DMEM supplemented with 20% charcoal-stripped fetal bovine serum. Twenty-four hours later BECs were treated for 24 h with either phenol red-free medium containing stripped fetal bovine serum alone (controls), or with 3.3×10^3^ pg/mL IL-1β, IL-6 or TNF-α. Following Tyrode wash, cells were incubated with an alternative P-gp substrate Rhodamine 123 (Rho123; 10^−5 ^M; Sigma) for 30 minutes. Rho123 was utilized to further validate the use of calcein-AM as an indicator of P-gp activity. Following lysis, Rho123 accumulation was measured (Ex/Em: 485/528 nm).

Cytokine effects on esterase activity were also assessed as we have described previously [19]. Calcein-AM is a non-fluorescing P-gp substrate, which in addition to extrusion by P-gp, is actively converted to fluorescent calcein by cytoplasmic non-specific esterases. To confirm that esterase activity was not affected by cytokines, BECs were treated for 24 h with either phenol red-free medium containing stripped fetal bovine serum alone (controls), or with 3.3×10^3^ pg/mL IL-1β, IL-6 or TNF-α. Cells were washed before the addition of warm lysis buffer containing 10^−6^ M calcein-AM. Conversion of calcein-AM to calcein, following treatment, was assessed immediately after 1-hour incubation with lysis buffer, as described above.

### Pro-inflammatory Cytokine Effects on Abcb1 mRNA Expression

*Abcb1* mRNA expression following cytokine treatment was only assessed in PND14 BECs as these cells exhibited the largest change in P-gp function. BECs derived from PND14 male guinea pigs were transferred to 0.5% gelatin-coated 10-cm culture dishes (Becton-Dickinson). At confluence, culture medium was removed and replaced with phenol red-free DMEM supplemented with 20% charcoal-stripped fetal bovine serum. Twenty-four hours following media change, cells were treated with 10^3^ and 3.3×10^3^ pg/mL of pro-inflammatory cytokines: IL-1β, IL-6 and TNF-α for 4 or 24 h at 37°C. These doses were chosen as they exhibited the largest change in P-gp function in PND14 BECs. Control cells were re-incubated with media containing charcoal-stripped fetal bovine serum. Following treatment, cells were placed on ice and washed twice with ice-cold Hank’s Balanced salt solution. RNA extraction was undertaken using TRIzol (Invitrogen), as previously described [5] and in accordance with the manufacturer’s instructions. Contaminating genomic DNA was removed by treating RNA samples with DNA-free deoxyribonuclease treatment (Ambion, Austin, Texas, USA). RNA purity and concentration were assessed using spectrophotometric analysis, and RNA integrity was verified using gel electrophoresis. RNA was stored at -80°C until further use. Total RNA was reverse-transcribed using High Capacity cDNA Reverse Transcription kit (Applied Biosystems, Carlsbad, California, USA). Samples were incubated at 25°C for 10 min, 37°C for 120 min, and 85°C for 5 min using the C1000 Thermal Cycler (Bio-Rad, Hercules, California, USA).

Real-time PCR was performed using the C1000 Thermal Cycler and quantified using the CFX96 Real-Time System (Bio-Rad). Samples were prepared using SsoFast EvaGreen Supermix (Bio-Rad), primer-probes for *abcb1*, *beta-actin* (*abcb1:* Forward – CAATCTGGGCAAAGATACTG, Reverse – CAAGTTCTTTGCTTTGTCCTC [Ensembl ID: ENSCPOT00000012540]; *beta-actin*: Forward – TTTACAATGAATTGCGTGTG, Reverse – ACATGATCTGGGTCATCTTC [Ensembl ID: ENSCPOT00000013600]), and cDNA template (100 ng) using ratios according to manufactures instructions. Data analysis was undertaken using *CFX Manager Software* (Bio-Rad). For each primer probe set, a standard curve was generated by serial dilution of a pooled reference sample with a minimum efficiency more than or equal to 90%. Samples were run in triplicate. Relative mRNA expression was calculated as gene of interest expression normalized [ΔΔc(t)] to reference gene expression (beta-actin). Beta-actin was not altered by pro-inflammatory cytokine treatment. For each plate, a non-template control (containing H2O in place of template cDNA) and non-amplification control (containing H2O in place of template RNA) was run to verify amplification and RT specificity.

### Statistical Analysis

All statistical analyses were performed using *Prism* (GraphPad Software Inc., San Diego, California, USA). Functional P-gp data as well as *abcb1* expression was analyzed using one-way ANOVA, followed by Dunnett’s (for comparisons against the control group). To assess baseline P-gp function across developmental ages, functional P-gp data was normalized against relative cell count and then expressed as percent change from GD40 BECs. For cytokine-treated BEC experiments, functional P-gp data is displayed as percent change in activity from controls. Significance was set at *P*<0.05.
